# ARSUIC Protocol: results in suicide prevention at a Mental Health Community Center

**DOI:** 10.1192/j.eurpsy.2025.2354

**Published:** 2025-08-26

**Authors:** R. Huerta-Ramírez, Á. Vivero

**Affiliations:** 1 Mental Health, Tajo University Hospital, Aranjuez (Madrid), Spain

## Abstract

**Introduction:**

Suicide has a growing importance as a mental health problem, being now the first cause of not-natural death at Spain. Developing suicide prevention strategies is a prioritary goal at mental health care. In our community, ARSUIC protocol was developed as a way to provide specialized preferent care to people with suicide attempts.

**Objectives:**

- Measure results of ARSUIC protocol at suicide attempts prevention.
- Study level of satisfaction of patients assisted at ARSUIC protocol.

**Methods:**

A qualitative ad-hoc phone interview has been made to patients atended at ARSUIC protocol of our community mental health center, in order to measure their level of satisfaction. Descriptive cuantification of new suicide attempts, visits to Emergency Service and incomings at Psychiatry Hospitalization in the last 6 months has been performed, using clinical history data.

**Results:**

Global level of satisfaction is high at the most of the sample, mainly because of the preferent attendance and the improvement it implies in the therapeutic bond. Indicators of relapse reflect global good evolution at the most of the sample.

**Conclusions:**

Preferent attendance reveals as a proper way to improve clinical care and prevention at people with suicide attempts. Strategies to maintain that kind of frequent attendance along the main risk period of relapse are in develope (group therapy, etc.).

**Disclosure of Interest:**

None Declared

